# Chemical signaling and insect attraction is a conserved trait in yeasts

**DOI:** 10.1002/ece3.3905

**Published:** 2018-02-14

**Authors:** Paul G. Becher, Arne Hagman, Vasiliki Verschut, Amrita Chakraborty, Elżbieta Rozpędowska, Sébastien Lebreton, Marie Bengtsson, Gerhard Flick, Peter Witzgall, Jure Piškur

**Affiliations:** ^1^ Department of Plant Protection Biology Swedish University of Agricultural Sciences Alnarp Sweden; ^2^ Department of Biology Lund University Lund Sweden; ^3^ Department of Agriculture and Food Science University of Applied Sciences Neubrandenburg Germany

**Keywords:** chemical signaling, Crabtree‐positive, fermentation, floral volatiles, insect attraction, mimicry, olfaction, pollination

## Abstract

Yeast volatiles attract insects, which apparently is of mutual benefit, for both yeasts and insects. However, it is unknown whether biosynthesis of metabolites that attract insects is a basic and general trait, or if it is specific for yeasts that live in close association with insects. Our goal was to study chemical insect attractants produced by yeasts that span more than 250 million years of evolutionary history and vastly differ in their metabolism and lifestyle. We bioassayed attraction of the vinegar fly *Drosophila melanogaster* to odors of phylogenetically and ecologically distinct yeasts grown under controlled conditions. Baker's yeast *Saccharomyces cerevisiae*, the insect‐associated species *Candida californica*,* Pichia kluyveri* and *Metschnikowia andauensis*, wine yeast *Dekkera bruxellensis*, milk yeast *Kluyveromyces lactis*, the vertebrate pathogens *Candida albicans* and *Candida glabrata*, and oleophilic *Yarrowia lipolytica* were screened for fly attraction in a wind tunnel. Yeast headspace was chemically analyzed, and co‐occurrence of insect attractants in yeasts and flowering plants was investigated through a database search. In yeasts with known genomes, we investigated the occurrence of genes involved in the synthesis of key aroma compounds. Flies were attracted to all nine yeasts studied. The behavioral response to baker's yeast was independent of its growth stage. In addition to *Drosophila*, we tested the basal hexapod *Folsomia candida* (Collembola) in a Y‐tube assay to the most ancient yeast, *Y. lipolytica,* which proved that early yeast signals also function on clades older than neopteran insects. Behavioral and chemical data and a search for selected genes of volatile metabolites underline that biosynthesis of chemical signals is found throughout the yeast clade and has been conserved during the evolution of yeast lifestyles. Literature and database reviews corroborate that yeast signals mediate mutualistic interactions between insects and yeasts. Moreover, volatiles emitted by yeasts are commonly found also in flowers and attract many insect species. The collective evidence suggests that the release of volatile signals by yeasts is a widespread and phylogenetically ancient trait, and that insect–yeast communication evolved prior to the emergence of flowering plants. Co‐occurrence of the same attractant signals in yeast and flowers suggests that yeast‐insect communication may have contributed to the evolution of insect‐mediated pollination in flowers.

## 1 INTRODUCTION

Yeasts are microscopic organisms, which communicate through a distinct aroma, rather than by visual signals. Even over distance, fermenting yeasts can be noticed by their characteristic, often sweet fragrance.

The smell of yeast consists of volatile metabolites produced during growth on organic substrates. A sweet smell is not exclusive for yeasts; it is an odor quality that we also attribute to flowers and fruits. Accordingly, many yeast‐derived volatiles are associated with aroma descriptors such as floral, flowery, and fruity (Lilly, Bauer, Styger, Lambrechts, & Pretorius, 2006; Lilly, Lambrechts, & Pretorius, 2000; Swiegers, Bartowsky, Henschke, & Pretorius, 2005).

Both flowers and fermenting yeasts attract foraging insects through emission of partially overlapping chemical signals (Stökl et al., 2010). The sweet odor of 2‐phenyl‐ethanol, for example, is a key constituent of the odor bouquets of yeasts and flowers. Not surprisingly, flower‐visiting insects can be lured to fermenting sugar substrates or sweet baits (El‐Sayed, Heppelthwaite, Manning, Gibb, & Suckling, 2005; Landolt, Todd, Zack, & Crabo, 2011).

Paleontology and molecular evolutionary biology suggest that yeasts, separating from filamentous fungi 300–400 mya (Dujon, 2006; Rolland & Dujon, 2011) and insects, being 300–400 million years old (Engel & Grimaldi, 2004; Nel et al., 2013) coexisted long before the origin of angiosperms, which evolved 125–150 mya (Clarke, Warnock, & Donoghue, 2011; Sun, Dilcher, Wang, & Chen, 2011). We hypothesize that emission of volatile signals and attraction of insects is an ancient and conserved trait in yeasts.

Interestingly, yeasts colonize flowers and volatiles produced by yeast can enhance floral signaling (Brysch‐Herzberg, 2004; Heiduk et al., 2017; Pozo, de Vega, Canto, & Herrera, 2009; Raguso, 2004; Schaeffer, Mei, Andicoechea, Manson, & Irwin, 2016). Pollination by insects existed already before the appearance of angiosperms (Labandeira, 2013; Labandeira & Currano, 2013) but the role of yeasts in the evolution of pollination is unknown.

Pre‐existing signals that mediated insect attraction to yeasts could have become beneficial for flowers by facilitating pollination. In view of overlapping chemical signals produced by flowers and yeasts, we discuss the concept that yeast‐like volatiles emitted by flowers or their associated microbes might have influenced the evolution of pollinator attraction. Clarifying the role of yeasts in insect attraction to flowering plants contributes to our understanding and possibly the management of plant–pollinator interactions, which is an urgent current challenge (Bailes, Ollerton, Pattrick, & Glover, 2015; Brown et al., 2016; Potts et al., 2016).

**Figure 1:**
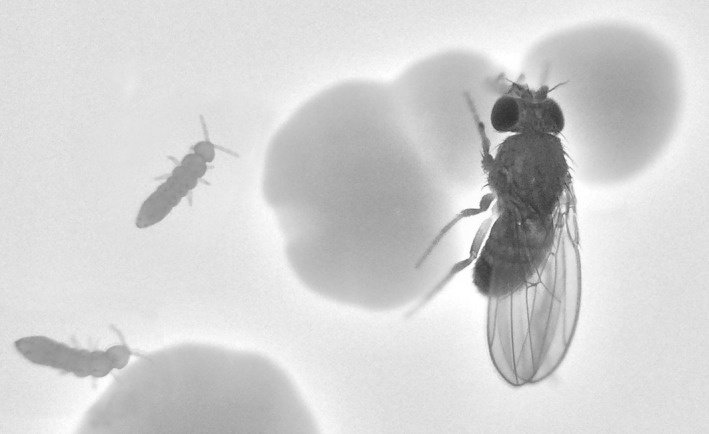
Two *Folsomia candida* springtails and a *Drosophila melanogaster* fly with colonies of *Saccharomyces cerevisiae* yeast

Yeasts spanning a wide ecological and phylogenetic range were grown under controlled growth conditions in bioreactors. Headspace samples of these yeasts were tested for bioactivity in the vinegar fly *Drosophila melanogaster* (Figure 1). Moreover, headspace of *Yarrowia lipolytica*, the most ancient yeast of our selection, was tested for attraction of the springtail *Folsomia candida* (Figure 1). Following chemical analysis of the headspace samples, a database search was conducted for co‐occurrence of yeast volatiles in flowering plants, and the behavioral role of these compounds in insect attraction. For the tested yeast species with known genome, we searched for the occurrence of genes involved in the synthesis of key volatiles. We conclude that insect‐yeast chemical communication is a phylogenetically ancient trait and suggest that yeasts may have played a role in the evolution of pollination.

## 2 MATERIAL AND METHODS

### 2.1 Yeast strains and cultivation

Nine yeast species that span a wide phylogenetic range were selected for investigating their capacity to attract *D. melanogaster* (Table 1). The selected species differ with respect to their distinct lifestyles and niches and include fruit associated species (*Saccharomyces cerevisiae, Dekkera bruxellensis*), human pathogens (*Candida albicans* and *Candida glabrata*), and species growing on milk products (*Kluyveromyces lactis*) or hydrocarbons (*Y. lipolytica*). The ecological attributes reflect specific adaptations in metabolism, such as anaerobic growth and the “make‐accumulate‐consume strategy” of the Crabtree positive *S. cerevisiae* and *D. bruxellensis* (Hagman, Säll, Compagno, & Piškur, 2013; Piškur, Rozpędowska, Polakova, Merico, & Compagno, 2006). Three species (*Candida californica, Metschnikowia andauensis*,* Pichia kluyveri*) are associated with insects (Table 1), two of them were isolated from *D. melanogaster* flies collected in a wine cellar (San Michele all'Adige, Italy). For isolation, *D. melanogaster* flies were trapped in sterile glassware and three single flies were transferred to YPD agar plates (yeast extract (Fluka), 1% w/v; peptone (Fluka), 2% w/v; D‐glucose (Sigma), 2% w/v; agar (Merck), 2% w/v). After 1 hr, flies were released from the three plates; plates were kept at ca. 20–25°C. One week later, three to four yeast colonies were isolated from each plate. Colonies were streaked separately on fresh YPD agar; purified yeast samples were preserved in 20% glycerol stock solutions at −78°C and identified as *C. californica* and *P. kluyveri* as described below. One isolate of each species was used for subsequent headspace analyses and fly assays. *M. andauensis* was isolated from an apple infested with a larva of *Cydia* pomonella (Witzgall et al., 2012).

**Table 1:** Specifications of the yeasts tested

Species name and strain designation^a^ (GenBankaccession)^b^	Laboratory designation at Lund University^c^	Doubling time t_d_/growth μ_max_ [hr ^−1^] at exp. growth	Optical density OD_max_	Isolation information	Characteristic
*Candida albicans* NRRL Y17967	Y1395	3.61/0.19	41.2	Unknown, Japan	Opportunistic human pathogen
*Candida californica* d(MG661810)	Y2131	2.42/0.29	24.8	*Drosophila melanogaster* from winery, Italy	Isolate from *D. melanogaster*
*Candida glabrata* CBS 138	Y1092	5.56/0.13	11.7	Feces of man	Opportunistic human pathogen
*Dekkera bruxellensis* CBS 2499	Y879	10.06/0.07	17.6	Wine	Fruit associated; causes wine spoilage; applied in breweries
*Kluyveromyces lactis* CBS 2359	Y707	2.21/0.31	26.53	Creamery	Industrial application e.g. dairies
*Metschnikowia andauensis*	Y2132	1.12/0.62	26.2	Gallery of *Cydia pomonella* in apple, Sweden	Fruit and insect associated
*Pichia kluyveri* e(MG661809)	Y2133	4.11/0.17	28.2	*D. melanogaster* from winery, Italy	Fruit associated
*Saccharomyces cerevisiae* CBS 8340	Y706	1.93/0.36	19.3	—	Industrial application e.g. winemaking, baking, brewing.
*Yarrowia lipolytica*	Y1688	2.59/0.27	22.5	—	Oleophilic, industrial application

a Abbreviations of culture collections—NRRL, The ARS Culture Collection, Peoria, IL, USA; CBS, Centraalbureau voor Schimmelcultures Utrecht Nl.

b Nucleotide accession at the GenBank database, National Center for Biotechnology Information (NCBI), Rockville Pike, USA, assigned to two yeast isolates from *Drosophila melanogaster*.

c Isolates are kept in stock at the Department of Biology at Lund University, Sweden.

d 99% Similarity with matched GenBank accession KY106378.1.

e 99% Similarity with matched GenBank accession KT156709.1.

Fresh colonies grown on YPD agar were applied for preparations of precultures in synthetic minimal medium as described by Verduyn, Postma, Scheffers, and Van Dijken (1992). Minimal medium was selected to focus on basic yeast volatiles, and to avoid volatile emissions from more complete or natural media typically emitting a strong smell. Controlled aerobic batch cultivation was performed in 1 L of medium using bench‐top bioreactors (Multifors, INFORS HT, Switzerland). Each reactor was inoculated with an approximately 500‐fold diluted starting culture compared to the biomass concentration in the bioreactors, at the end of each experiment (Hagman et al., 2013). The cultivation was maintained at 25°C and aerated with an airflow of 1 L/min, dissolved oxygen was measured, and concentration >30% of saturation was maintained by regulation of steering; a pH of 5 was maintained by automatic pH measuring and buffering with 1M H_2_SO_4_ and 2M KOH. All yeasts showed exponential growth and increase in biomass (Table 1). Cultivations were allowed to grow until reaching maximum cell density in the stationary phase. Yeast growth was followed by continuous measurement of CO_2_ and O_2_ concentrations in the gas outlet, and regular measurement of OD in the suspension. Samples of 50 ml volume were taken every 3–4 hr during the cultivation of the individual yeasts. Fermentation was stopped by immediately cooling down the samples to −80°C. For each of the yeasts, the sample representing the highest cell density before reaching the stationary phase was selected for behavioral analysis. Additional samples of earlier growth phases with lower cell densities were selected from the cultivation of *S. cerevisiae*.

### 2.2 Yeast taxonomic identification

Ten colonies isolated from three wild *D. melanogaster* flies were cultivated for analysis of species identity. For genomic DNA extraction, 4 ml of liquid YPD medium was inoculated with single yeast colonies and incubated overnight at 25°C. The cultures were spun down (10,060 *g*, 2 min) and washed with water. 200 μl of the lysis buffer (2% Triton X‐100, 1% SDS, 0.1 mol/L NaCl, 0.001 mol/L EDTA, 0.01 mol/L Tris at pH 8), 200 μl phenol:chloroform:isoamyl alcohol (25:24:1), and 100 μl acid‐washed glass beads were added to the pellet. The mix was vortexed for 10 min, and 200 μl of TE buffer (10 mmol/L Tris at pH 7.5 – 8, 1 mmol/L EDTA at pH 8) was added. The suspension was centrifuged for 10 min at 10,060 *g*, and 10 μl RNase A (10 mg/ml) was added to the aqueous phase and incubated for 45 min at 37°C. The DNA was precipitated with 96% ethanol and 3 mol/L sodium acetate. The mixture was centrifuged for 10 min at 10,060 *g* at 4°C. The pellet was washed with ice‐cold 70% ethanol, air‐dried, and re‐suspended in 40 μl TE buffer (pH 8).

For each isolate, the D1/D2 domain of the 26S ribosomal DNA region was amplified using the NL1 (5′‐GCATATCAATAAGCGGAGGAAAAG‐3′) and NL4 (5′‐GGTCCGTGTTTCAAGACGG‐3′) primers (Kurtzman & Robnett, 1997). The PCR reaction was performed at an initial denaturation at 95°C for 5 min, followed by 30 cycles at 95°C for 30 s, 52°C for 30 s, 72°C for 1 min with a final extension at 72°C for 10 min. Five microliters of PCR products were analyzed using 1.0% agarose gel electrophoresis. The gel was stained with GelRed nucleic acid stain (Biotium Inc.) and visualized under UV light. The PCR products were then purified and sequenced using the BigDye Terminator v3.1 Cycle Sequencing kit (Applied Byosystems) in an ABI 3730 automated sequencer (Applied Biosystems). The sequences obtained were aligned with the GenBank database sequences using the basic local alignment search tool (BLAST) at the National Center for Biotechnology Information (Altschul, Gish, Miller, Myers, & Lipman, 1990). Sequences of the two identified species were submitted to the GenBank database and designated with accession numbers (Table 1).

### 2.3 Headspace sampling and chemical analysis

We modified the gas outlet system of the bioreactors by inserting air filters (Super Q, 80/100 mesh; Alltech, Deerfield, IL, USA) to collect volatiles emitted by the fermenting yeast cultures. Online sampling from the bioreactors was aiming at collecting odors at specific stages of the fermentation process rather than a blend of odors emitted during changing growth conditions. Adsorbed headspace volatiles were eluted from the air filters with heptane and methanol. Eluents corresponding to the samples selected for behavioral analysis were analyzed by GC‐MS (6890 GC and 5975 MS, Agilent Technologies Inc., Santa Clara, CA, USA). Two μl of sample were injected spitless (injector temperature 225°C) into a DB‐wax column (30 m × 0.25 mm × 0.25 μm film thickness; J&WScientific, Folsom, CA, USA). The GC temperature program was 30°C (3 min) and 8°C/min to 225°C (5 min). Helium was used as mobile phase at 35 cm/s. The MS was operated in the electron impact mode with the electron energy set at 70 eV and a scan range over 29–400 m/z.

In addition, solid phase microextraction (SPME) with a divinylbenzene (DVB)/Carboxen (CAR) on polydimethylsiloxane (PMDS) coating, 50/30 μm coating × 1 cm, Supelco, USA was performed from biomass samples tested in the wind tunnel to specifically check for volatile compounds not recorded by the GC‐MS (solvent delay) when analyzing Super‐Q filter eluates. For SPME, yeast samples of 50 ml were kept in 100‐ml Erlenmeyer flasks closed with aluminum foil. The fiber was conditioned for 20 min in a GC injection port at 270°C, passed through a small hole in the foil and exposed above the yeast sample. After 10 min of sampling, the fiber was immediately subjected to GC‐MS analysis under similar settings as described above. Headspace compounds were identified according to retention indices and mass spectra, in comparison with a reference library (NIST, Agilent) and authentic reference compounds.

Every few hours, samples of 2 ml were taken from the fermentors for detailed analysis of metabolites produced during the fermentation of *S. cerevisiae*. Concentrations of glucose, ethanol, acetate, and glycerol were determined with a HPLC 1200 series (Agilent) equipped with a 300*7.7 mm Aminex HPX‐87H Column (Bio‐Rad). The mobile phase was H_2_SO_4_ (5 mmol/L), and flow rate was set to 0.6 ml/min. Column temperature was set to 60°C and RID temperature to 55°C (Hagman et al., 2013).

### 2.4 Wind tunnel assay

A flight tunnel (30 × 30 × 100 cm^3^) was used to test attraction of *D. melanogaster* (Dalby‐HL) to yeast headspace (Becher, Bengtsson, Hansson, & Witzgall, 2010). Within 12 hr after emergence, flies were transferred from vials with fly food to vials containing damp cotton only (i.e., flies were starved). Flies were not sexed and not controlled for mating state. The flies were kept similar to rearing conditions under a 12:12‐hr L/D photoperiod and tested 2 days later, 2–4 hr after beginning of the photophase. For each yeast, five batches of twenty flies were tested for upwind flight against an air stream of 0.25 m/s and landing at the odor source. Flies were released from a jar (Becher et al., 2010) at the downwind end of the tunnel and exposed to yeast odor delivered from the upwind end. For odor delivery, yeast samples were brought to room temperature and transferred to a wash bottle prior to testing. Charcoal filtered air (0.5 L/min) was blown through the bottle and, via an attached Pasteur pipette, yeast volatiles were injected into a glass jar in the center of the wind tunnel at the upwind end (Becher et al., 2010). Air blown through minimal medium was used for control. Landing on the Pasteur pipette, the rim of the jar or inside the jar was scored during a test period of 15 min (Becher et al., 2010). Wind tunnels allow discriminative and sensitive testing as flies fly upwind only in response to a behaviorally relevant stimulus. Flies discriminate odor quality as well as quantity, and flies respond in accordance with their internal physiological state; testing flies individually or in batches both allows discriminative testing (Becher et al., 2010; Lebreton et al., 2015). Landing behavior, scored in this study, is the most stringent criterion of measuring odor‐mediated upwind flight attraction.

### 2.5 Y‐tube assay

A bioassay using a Y‐tube was conducted to test the odor of the most ancient yeast in our study, *Y. lipolytica*, for attraction of the collembolan *F. candida* (Terra‐Jungle, Germany) as a representative of basal noninsect hexapods. Springtails were 2–3 weeks old and starved on humidified plaster of Paris for 24 hr before the assay. All tested individuals (parthenogenetic females) were of similar size (ca. 2 mm long). The Y‐tube system was based on the olfactometer described by Bengtsson, Hedlund, and Rundgren (1991) with slight modifications. Side arms (40 mm long) of the Y‐tube (8 mm inner diameter) were connected to glass tubes where a suspension of *Y. lipolytica* or growth medium for control was applied on filter paper. Charcoal filtered air (12 ml/min) was sucked through the system using a pump connected to the stem (30 mm long) of the Y‐tube. Springtails were tested individually for their first choice of yeast vs. medium (entering one of the arms) during a test period of 5 min, under low light conditions at 25°C and 60 ± 5% relative humidity.

### 2.6 Blast search

After behavioral and chemical studies of yeast volatiles, we were interested if the ability to produce certain compounds was reflected in published yeast protein sequences. BLAST search for protein sequences from six yeast genomes was conducted to identify orthologous genes involved in the synthesis of ethyl acetate (ATF1, ATF2), in transamination of amino acids (ARO8, ARO9, BAT1, BAT2) and reduction of aldehydes (ALD2, ALD3, ALD4, ALD5, ALD6) to fusel alcohols like 3‐methyl‐1‐butanol, 2‐methyl‐1‐butanol, and 2‐phenylethanol and fusel acids like 2‐phenylacetate, and in the formation of acetoin (BDH1, BDH2) (González et al., 2010; Hazelwood, Daran, van Maris, Pronk, & Dickinson, 2008; Verstrepen et al., 2003). Genes of *S. cerevisiae* (S288c) were used for a fungal BLASTP against *Y. lipolytica* (CLIB122), *C. albicans* (WO‐1), *D. bruxellensis* (AWRI 1499), *K. lactis* (Y707), *C. glabrata* (CBS 138), and *S. cerevisiae* (Y 706). All reciprocal best hits (E‐values <10^−4^_,_ identities >25%, similarities >30%) for each gene and species are summarized in Appendix S1.

### 2.7 Data sources

Data on the occurrence of specific floral volatiles were obtained from the Pherobase (El‐Sayed, 2016). Noteworthy, floral emissions of volatiles generally contain a number of metabolites produced by microbes associated with flowers but studies discriminating between plant‐ and microbe‐derived metabolites are rare (Lenaerts et al., 2017). The Pherobase was also consulted for behavioral activity of floral odors. The Saccharomyces Genome Database (SGD) using WU‐BLAST2 was consulted for the reciprocal BLAST search.

## 3 RESULTS

### 3.1 Identification of yeasts isolated from *Drosophila* flies

Yeasts isolated from *D. melanogater* flies were identified as *C. californica* (six isolates) and *P. kluyveri* (four isolates). For each species, we submitted the nucleotide sequences of one isolate to GenBank for assignment of accession numbers (Table 1).

### 3.2 All yeasts tested attract *Drosophila* flies

**Figure 2:**
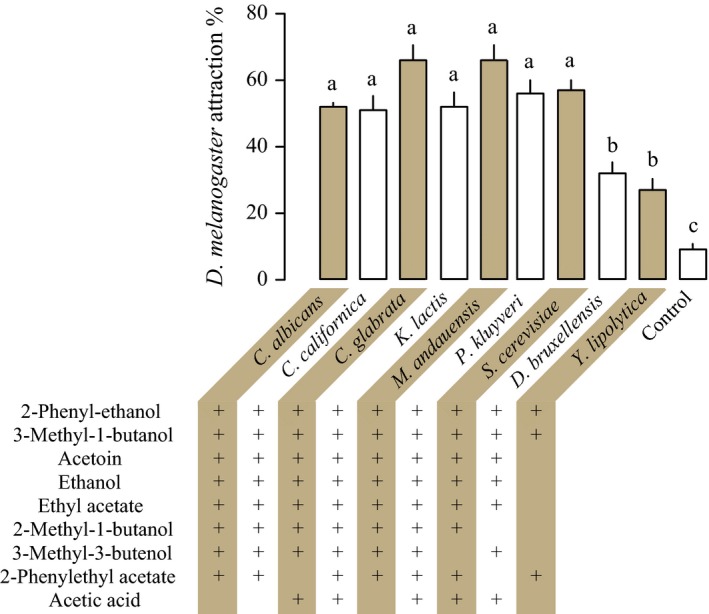
Upwind flight attraction of *Drosophila melanogaster* flies followed by landing at the odor source in response to headspace volatiles of nine yeast species. Yeasts were grown as controlled aerobic batch culture on synthetic minimal medium. All yeasts induced significant attraction behavior (ANOVA,* F *= 25.47, *df *= 49, *p* < .0001; different lower case letters indicate significant difference). Predominant volatiles repeatedly identified in yeast headspaces are shown (+)

Evolutionarily distant and ecologically distinct yeast species were cultivated to test for their ability to attract flies in a wind tunnel assay. Headspace of all yeasts tested during the late exponential growth phase elicited a significant behavioral response in *D. melanogaster* (ANOVA, *F *= 25.47, *df *= 49, *p *< .0001; *n *= 5; Figure 2).

Odor‐mediated upwind flight and landing at the odor source was significantly stronger toward *S. cerevisiae, K. lactis, C. glabrata, P. kluyveri, C. californica, M. andauensis,* and *C. albicans* than toward *D. bruxellensis* and *Y. lipolytica* (Tukey's multi comparison test, *p* < .05; Figure 2).

### 3.3 Yeasts attracting *Drosophila* share volatile compounds

Yeasts grown under controlled conditions were used for behavioral and chemical analyses. During short sampling intervals, corresponding to the duration of behavioral tests, we found nine compounds that repeatedly occurred in at least five of the nine yeasts. Two compounds, 2‐phenyl‐ethanol and 3‐methyl‐1‐butanol, were released by all nine yeasts. Acetoin, ethanol, and ethyl acetate were detected in all but one yeast, *Y. lipolytica* (Figure 2).

In *S. cerevisiae*, alcohol acetyl transferase catalyses the synthesis of acetate esters (Verstrepen et al., 2003). In agreement with the absence of ethyl acetate in *Y. lipolytica* headspace, ATF1 and ATF2 were not found in the published genome of *Y. lipolytica* (CLIB122) (Appendix S1). Moreover, ATF1 and ATF2 could not be found in the published genomes of *D. bruxellensis* (AWRI 1499) and *C. albicans* (WO‐1).

Butanediol dehydrogenase catalyzes the synthesis of acetoin in *S. cerevisiae* (González et al., 2010). Corresponding to the lack of acetoin in *Y. lipolytica* headspace, BDH1 and BDH2 were absent in *Y. lipolytica* (CLIB122).

Fusel compounds are fermentation products derived from amino acid catabolism via the Ehrlich pathway (Hazelwood et al., 2008). All yeasts were producing the fusel alcohols 3‐methyl‐1‐butanol, as a typical catabolite of the branched amino acid leucine, and 2‐phenyl‐ethanol, derived from the aromatic tryptophane. Each of the yeast genomes contained at least one of the examined orthologs encoding aldehyde dehydrogenases (ALD2‐6), aromatic aminotransferases (ARO8‐9), and branched‐chain aminotransferases (BAT1‐2), being enzymes of the Ehrlich pathway (Appendix S1). Overall, for the 13 investigated genes, *S. cerevisaie* had five orthologs in common with the earliest diverging yeasts *Y. lipolytica*, and 10 with the more closely related yeast *K. lactis* (Appendix S1).

### 3.4 *Drosophila* attraction is independent of yeast growth stage

**Figure 3:**
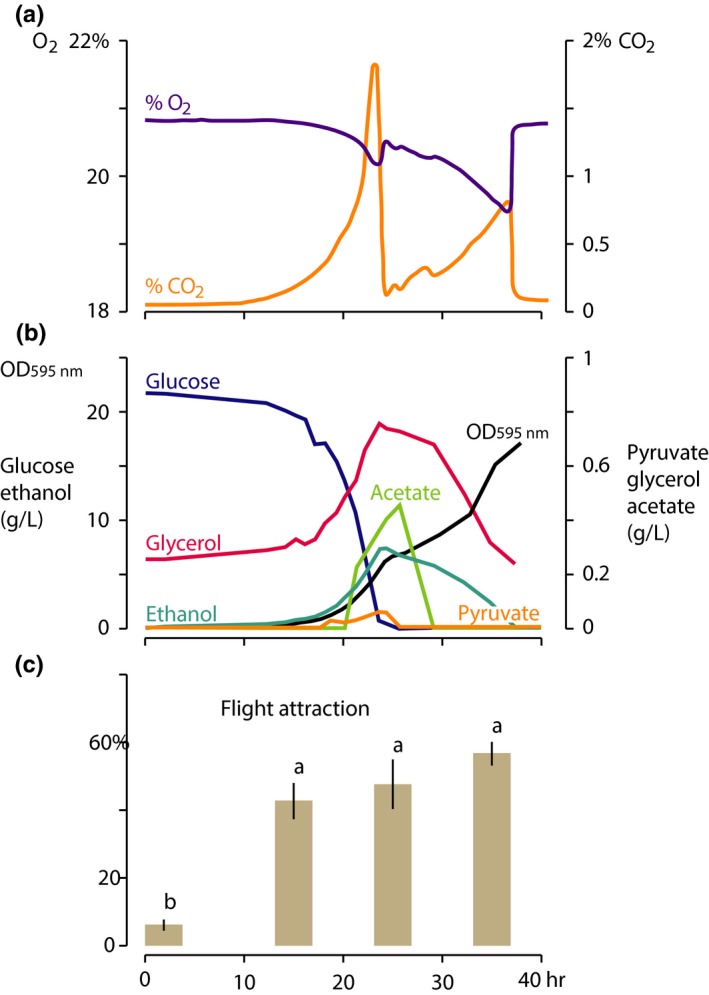
Growth of *Saccharomyces cerevisiae* under controlled conditions and attraction of *Drosophila melanogaster* toward yeast volatiles emitted before and after the metabolic shift. A change of O_2_ and CO_2_ concentrations (a), cell growth (illustrated by increasing optical density OD at 595 nm), and fermentation products (b) shows the shift from aerobic glucose fermentation to respiration at about 23.4 hr after inoculation of the cultivation. (c) Flies were similarly attracted toward odors emitted at 14.2 hr, 25.8 and 35 hr after inoculation, while significantly fewer flies (ANOVA,* F *= 35.03, *df *= 24, *p *< .0001; different lower case letters indicate significant difference) were attracted to freshly inoculated mineral medium that was similar to control (6 ± 2.5%, not shown)

Crabtree‐positive yeasts like *S. cerevisiae* aerobically ferment sugar to ethanol and, after depletion of sugar, switch from respiro‐fermentative growth to strict respiration and degradation of earlier produced ethanol (Piškur et al., 2006). We tested whether *D. melanogaster* responds similarly to odors emitted during respiro‐fermentative and respiratory metabolism. The sudden drop of CO_2_ shows the diauxic shift (23.4 hr after inoculation, Figure 3a). This shift is accompanied by a distinct short lag phase followed by respiration and ethanol assimilation and a decrease in ethanol and glycerol concentration in the medium (Figure 3a,b).

Upwind flight and landing behavior toward *S. cerevisiae* sampled at different times of the controlled batch fermentation showed that fly attraction was independent of growth stage (Figure 3c). Attraction to headspace samples collected during the exponential, respiro‐fermentative growth phase on glucose (14.2 hr after inoculation) and during the early, respiratory growth phase on ethanol (25.8 hr after inoculation) was high and similar as to samples collected during late respiratory growth (35 hr after inoculation), when ethanol concentrations and growth had declined (ANOVA, *F *= 35.03, *df *= 24, *p *< .0001 *n *= 5). In contrast, attraction to the headspace emitted directly after inoculation (0 hr) was low and similar to control (Figure 3c). Similar as for ethanol, the concentration of acetate being in dissociation with acetic acid (pKs = 4.75) was decreasing after the diauxic shift. High concentrations of ethanol and acetate (25.8‐hr samples) did not increase fly attraction (Figure 3b,c).

### 3.5 Yeast attracts springtails

Volatiles produced by the phylogenetically most ancient yeast, *Y. lipolytica*, were tested for attraction of collembolans, early terrestrial noninsect hexapods. A significant number (70%; C.I. = 0.51–0.84) of *F. candida* springtails were attracted to odor emissions of *Y. lipolytica* when tested against a medium control in the Y‐tube olfactometer (Exact Binomial Test, *p *< .05, *n *= 33).

### 3.6 Yeast volatiles are common floral signals and insect attractants

**Figure 4:**
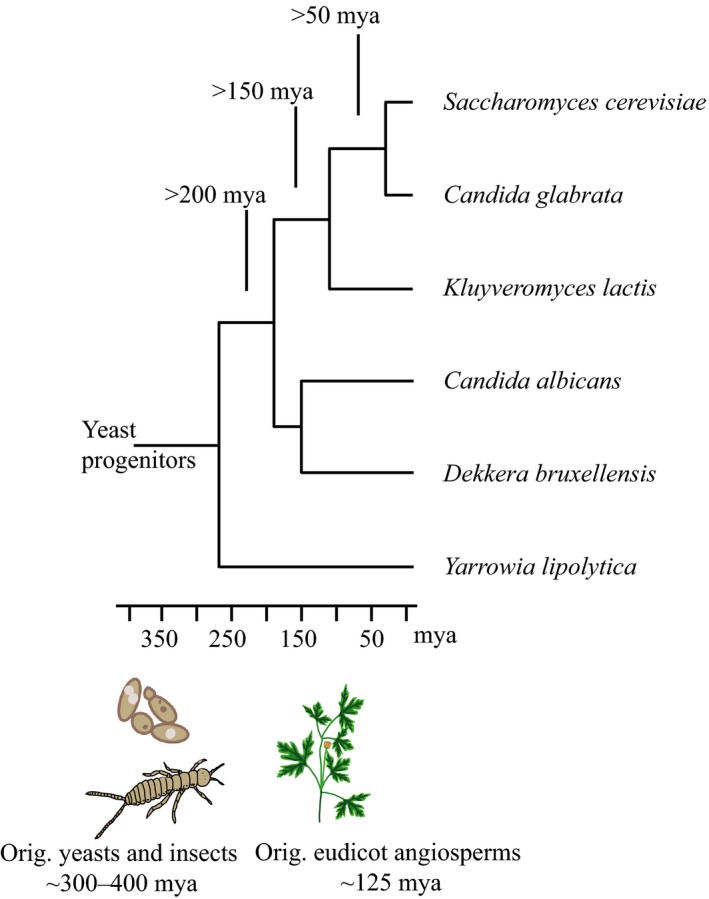
Simplified phylogenetic tree of yeasts studied here. The phylogenetic position of *Candida californica, Metschnikowia andauensis,* and *Pichia kluyveri* (not shown) is unclear, but these are more closely related to *Saccharomyces cerevisiae* than *Yarrowia lipolytica*. Insect–yeast interactions predate the evolution of flowering plants

**Figure 5:**
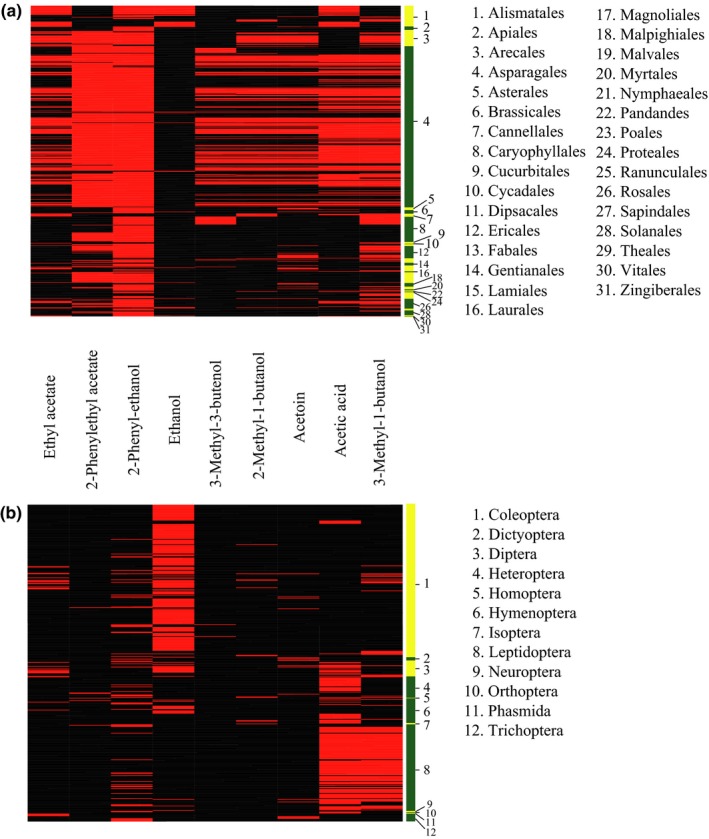
(a) The presence (red) or absence (black) of selected yeast‐like volatiles in 700 flowering plants (angiosperms) from 31 orders. Volatiles were selected according to their presence in yeast headspace. (b) Behavioral activity (red) in 479 insect species of 12 orders toward selected yeast‐like volatiles emitted from angiosperms. Inactivity of volatiles (black) means that no behavioral activity of the compound is known for a certain insect species. The green and yellow coding of the sidebars alternates for 31 plant (a) and 12 insect (b) orders, and the length of the green or yellow sections illustrates the abundance of species within those orders. Data were extracted from the Pherobase (El‐Sayed, 2016)

Volatile signals of phylogenetically distant yeasts spanning a period of several 100 million years of species diversification (Figure 4) mediate insect attraction (Figure 2). Literature and database revision revealed that yeasts and flowers share volatile compounds (Figure 5a), which are attractive to insects (Figure 5b). Our review shows that 700 flowering plants of 31 orders were described to emit at least one of the nine volatiles repeatedly found in the studied yeasts. Orchids (Asparagales) have most frequently been reported to emit yeast volatiles. Behavioral activity toward the volatiles emitted by flowering plants has been described in 479 insects from 12 orders. Among these, most records of insects responding to yeasty floral volatiles concerned coleopteran and lepidopteran species.

## 4 DISCUSSION

### 4.1 Insect attraction is a common and conserved trait in yeasts

Yeasts produce volatile chemical signals that attract insects (Andreadis, Witzgall, & Becher, 2015; Davis, Crippen, Hofstetter, & Tomberlin, 2013; Mori et al., 2017; Witzgall et al., 2012). Early species of yeasts and insects have coexisted for more than 300 my and coevolution of chemical signals and chemosensory systems facilitates odor‐mediated interactions between yeasts and insects (Dujon, 2006, 2010; Engel & Grimaldi, 2004; Nel et al., 2013; Scheidler, Liu, Hamby, Zalom, & Syed, 2015).

Yeast strains and species are polymorphic with respect to volatile production (Arguello, Sellanes, Lou, & Raguso, 2013; Scheidler et al., 2015). However, after controlled fermentation on a synthetic minimal medium, all species in this study induced attraction of flies. Medium containing just the minimal necessities for yeast growth is sufficient to produce cultures that attract flies; we expect that fermentation of more complex or natural media would lead to richer bouquets of odors (Swiegers et al., 2005) and possibly even higher insect attraction. Recent studies (Buser, Newcomb, Gaskett, & Goddard, 2014; Palanca, Gaskett, Günther, Newcomb, & Goddard, 2013) showed significant fly attraction toward yeast‐produced volatiles (emitted from fermented YPD or grape juice) with the level of attractiveness depending on yeast species or strain, respectively.

Attraction of *Drosophila* to yeasts not associated with fruit or insects was expected to be lower than attraction toward yeasts ecologically linked to host fruit or insects (Palanca et al., 2013). However, our study suggests that production of volatiles that attract insects is a conserved trait, which embraces yeasts of various habitats, including vertebrate pathogens. Furthermore, flies were attracted to yeasts differing in their physiological characteristics of sugar metabolism and, moreover, to *S. cerevisiae* at different growth phases, suggesting that attraction is a common trait and not limited to a specific type of yeast metabolism.

### 4.2 Yeast volatiles promote communication with insects

Odorants facilitate recognition of yeasts, fruit, and flowers even from distance (Buser et al., 2014; Palanca et al., 2013; Raguso, 2004; Saveer et al., 2012). Fermenting fruit such as apples or grapes generally have a richer and more intensive odor profile than fresh fruit due to the yeast‐derived volatile fraction (Swiegers et al., 2005). For *D. melanogaster,* yeast‐derived volatiles are behaviorally more important than fruit compounds (Becher et al., 2012).

Coadaptation between yeast volatile emission and insect olfaction most likely underlies ecological relations between yeasts and *Drosophila* (Scheidler et al., 2015). Core metabolic processes in *S. cerevisiae* mediate the production of volatile signals attracting *D. melanogaster* (Schiabor, Quan, & Eisen, 2014).

Numerous studies report that insects vector yeast internally and externally of their body, for example, *D. melanogaster* (Becher et al., 2012; Chandler, Eisen, & Kopp, 2012; Christiaens et al., 2014; Gilbert, 1980; Stamps, Yang, Morales, & Boundy‐Mills, 2012; Starmer & Fogleman, 1986). More attractive strains of *S. cerevisiae* are more likely dispersed by *D. simulans* or other insects (Buser et al., 2014). Interestingly, the yeasts we isolated from *D. melanogaster* flies trapped in an Italian winery split into *P. kluyveri* and *C. californica*, representing two of the three species that consistently formed yeast communities with *D. melanogaster* larvae on banana (Stamps et al., 2012). Together with another described isolation of *C. californica* from *D. melanogaster* (Stötefeld, Holighaus, Schütz, & Rohlfs, 2015), these findings support the existence of species‐specific adaptations between *D. melanogaster* and yeasts.

In addition to the benefit of substrate‐directed vectoring, *S. cerevisiae* spores benefit from passing the fly gut, which increases dispersion and outbreeding (Pulvirenti, Zambonelli, Todaro, & Giudici, 2002; Reuter, Bell, & Greig, 2007). Moreover, fruit infested with *D. melanogaster* develops higher yeast densities than fruit without larvae (Stamps et al., 2012) and larval feeding reduces growth of mold (Hodge, Mitchell, & Arthur, 1999; Rohlfs, 2005; Wertheim, Marchais, Vet, & Dicke, 2002).

Yeast volatiles mediate attraction of vectors and seem to be less important for functions of cell viability. The ATF1 gene in *S. cerevisiae* encodes an alcohol acetyl transferase responsible for ester formation and was shown to promote attraction of *D. melanogaster* (Christiaens et al., 2014). However, ATF1 is not essential for cell survival and plays no known metabolic function apart from the formation of acetyl esters. A BLAST search revealed the absence of ATF1 and ATF2 in the published genome of *Y. lipolytica* (CLIB122), which corresponds to a lack of detectable amounts of ethyl acetate and lower fly attraction in the wind tunnel, in comparison with other yeasts tested, except *D. bruxellensis* (Figure 2; Appendix S1). Indeed, ATF1 and ATF2 also are absent in *D. bruxellensis* (AWRI 1499), although ethyl acetate was detected in our headspace analysis of *D. bruxellensis* (CBS 2499). This compares to reduced, but detectable production of ethyl acetate in *S. cerevisiae* ATF deletion strains (Christiaens et al., 2014; Verstrepen et al., 2003).

The presence of additional orthologs of genes related to the production of acetoin or fusel compounds in all yeasts included in our BLAST search, together with our behavioral and chemical analysis, supports the view that production of volatile signals is conserved and that other fermentation products in addition to acetate esters contribute to insect attraction. In addition to evolutionary conservatism, also convergent evolution might contribute to similarity in chemical signals (Bohlmann, Meyer‐Gauent, & Croteau, 1998; Courtois et al., 2016).

Several compounds found in yeast headspace are by‐products of cell metabolic processes like carbohydrate and protein metabolism (Albertazzi, Cardillo, Servi, & Zucchi, 1994; Hazelwood et al., 2008; Lilly et al., 2006; Piškur et al., 2006) and chemical signaling by volatiles might have developed as a secondary function of emitted metabolic products. Additional functions like inhibition of competitive microorganisms by volatiles are likely and could affect compound release and fitness (Hua, Beck, Sarreal, & Gee, 2014; Piškur et al., 2006).

### 4.3 Yeast coexistence wth insects predates coevolution between insects and flowers

Flies were strongly attracted to yeasts, disregarding their taxonomic position. Five of the examined species contained 2‐phenyl‐ethanol, acetic acid, acetoin, and 3‐methyl‐1‐butanol, previously shown to induce strong upwind flight attraction in *Drosophila* (Becher et al., 2012). 2‐Phenyl‐ethanol, present in all yeasts, was the main volatile in headspace of *Y. lipolytica,* which in our study was the most ancient yeast. 2‐Phenyl‐ethanol is a key component of fermentation odor blends attractive to *D. melanogaster* (Becher et al., 2010, 2012; Zhu, Park, & Baker, 2003).

An early origin of yeast chemical signaling and the ability to attract potential vectors was further confirmed by attraction of the basal hexapod *F. candida* to *Y. lipolytica* (Regier, Shultz, & Kambic, 2004). Similar to insect–yeast interactions, attraction of collembolans to yeast volatiles likely is based on trophic interactions and vector‐mediated microbial dispersal (Men'ko, Chernov, & Byzov, 2006; Thimm, Hoffmann, Borkott, Munch, & Tebbe, 1998). Interestingly, springtails vector sperm of mosses and possibly represent an early form of animal‐mediated fertilization in terrestrial plants (Cronberg, Natcheva, & Hedlund, 2006).

Coexistence with plants and animals has influenced chemical processes and ecology in fungi since the Paleozoic (about 550 mya), allowing the establishment of new associations with plants and animals (parasitism, symbiosis) involving co‐evolutionary processes (Brundrett, 2002; Taylor & Berbee, 2006; Taylor & Osborn, 1996). Budding yeasts (hemiascomycetes) as major taxonomic group probably separated from filamentous fungi at the latest 300–400 mya (Dujon, 2006, 2010; Heckman et al., 2001) and have adapted to specialized niches where they typically exploit substrates rich in organic carbon. Ancestral wingless hexapods (Collembola, Protura, Diplura) and insects evolved during the same period as budding yeasts. By the Pennsylvanian, ca. 320 mya, pterygot insects were present, including further derived holometabolous insects (Engel & Grimaldi, 2004; Faddeeva et al., 2015; Knecht, Engel, & Benner, 2011; Misof et al., 2014; Nel et al., 2013).

Insect olfactory neurons express three types of chemoreceptors, ionotropic receptors (IRs), gustatory receptors (GRs), and odorant receptors (ORs). Recent work suggests that ORs, being younger than GRs and IRs, evolved in pterygot insects and increased the detection spectrum of compounds but also sensitivity and speed of detection, which is important for odor‐sensing during flight (Croset et al., 2010; Getahun, Wicher, Hansson, & Olsson, 2012; Missbach et al., 2014). The evolution of ORs would thus coincide with the evolution of early yeasts, and several yeast volatiles are indeed known as OR ligands (Münch & Galizia, 2016).

Yeast hyperdiversity in insect guts (Boekhout, 2005; Suh, McHugh, Pollock, & Blackwell, 2005) is one aspect reflecting the ecological significance of the diversification of insects for the evolution of yeasts. Triassic amber samples, 230 my old, show the presence of flies, bacteria, and microfungi (Schmidt et al., 2012). Finally, with eudicot angiosperms being widely distributed in early Cretaceous by about 125 mya ago, the production of fruit provided unparalleled access to sugar (Sun et al., 2011), and the development of new growth strategies in *Saccharomyces* yeasts (Piškur et al., 2006), leading to fruit‐associated yeast–insect interactions.

### 4.4 Parallels to the pollination concept

Smell the yeasts. Their odors are pleasant and sweet. Yeasts and flowers share volatile signals (Figure 5a) which are attractive to insects (Figure 5b). The ecological role of such volatiles is well established for flowers but not for yeasts: the metaphoric title “Wake up and smell the roses” (Raguso, 2008) emphasizes the importance of volatiles that had not sufficiently been acknowledged in pollination biology. Most angiosperms require pollinators for reproduction (Schoonhoven, van Loon, & Dicke, 2005) and floral volatiles mediate pollinator attraction (Figure 5b). There is a clear functional analogy between yeast spores and flower pollen, and insects mediate dispersal as well as outbreeding in both. In return, insects benefit from their visit through a food reward (Knauer & Schiestl, 2015; Yamada, Deshpande, Bruce, Mak, & Ja, 2015).

Pollinator attraction by fungi for the purpose of spore dispersal has been described for ascomycete and basidiomycete fungi (Kaiser, 2006; Roy, 1993; Roy & Raguso, 1997; Schiestl et al., 2006). Furthermore, it was proposed that convergent development has led to the evolution of chemical insect attractants in fungi and plants (Schiestl et al., 2006) and to floral mimicry of decaying plant or animal material (Jürgens, Wee, Shuttleworth, & Johnson, 2013).

Vectoring of yeast likely predates vectoring of pollen (Figure 4) and signals mediating insect–yeast interactions possibly facilitated the attraction of insect pollinators already in ancient angiosperms. Emission of pre‐existing insect attractants could have been beneficial by increasing floral pollination and fitness. Yeast‐like floral volatiles have been described for archaic angiosperms. 2‐Phenyl‐ethanol is a dominant compound in the relictual‐basal angiosperm *Trimenia moorei*, considered as pollinator attractant and found in additional basal angiosperms that are visited by pollen‐vectoring insects (Bernhardt et al., 2003; Thien et al., 2009). Flowers of the relictual angiosperm *Zygogynum bicolor* (Winteraceae) emit a “musty” smell and are pollinated by ancient micropterigid moths of the genus *Sabatinca*; the association between *Zygogynum* and *Sabatinca* is suggested to exist since the early evolution of flowering plants (Pellmyr & Thien, 1986; Thien et al., 1985). Flowers of *Z. bicolor* and two other species of winteraceae emit ethyl acetate as main volatile and 2‐methyl‐1‐butanol and acetic acid to a minor content (Thien et al., 1985).

Yeasts are commonly found in flowers and floral nectar and their volatile emissions might directly mediate plant signaling and pollinator attraction (Lachance & Bowles, 2002; Pozo, Lievens, & Jacquemyn, 2014; Pozo et al., 2009). Yeast‐like volatiles emitted by flowers or their associated microbes consequently might have influenced the evolution of pollinator attraction. We propose the emission of ancient, pre‐existing microbial signals as a component of the evolution of pollination in flowering plants and suggest investigating the role of yeasts to complement existing concepts on the evolution of pollination (Jürgens et al., 2013; Pellmyr & Thien, 1986; Schiestl et al., 2006).

Likewise, Schiestl and Dötterl (2010) suggested that floral volatile evolution was driven by pre‐existing sensory preference for “floral‐like” signals produced and detected by insects. Olfactory mimicry of fermentation odors was previously shown for the lily *Arum palaestinum* (Stökl et al., 2010). Volatiles we found in yeast headspace (Figure 5a) were most prominent in orchids (Asparagales), which are known to deceive pollinators by mimicking floral signals or pollinator sex pheromones (Heiduk et al., 2017; Jürgens et al., 2013; Schiestl et al., 1999; Stökl, Brodmann, Dafni, Ayasse, & Hansson, 2011).

In summary, we studied a phylogenetically broad range of hemiascomycetous yeasts framed by the alkane‐utilizing *Y. lipolytica* and the sugar degrading Crabtree‐positive *S. cerevisiae*. These species and their volatile signals most likely were present when *D. melanogaster* and other close related species within the *melanogaster* subgroup appeared less than 50 mya (Ometto et al., 2013; Wiegmann, Yeates, Thorne, & Kishino, 2003; Wiegmann et al., 2011). As *D. melanogaster* is attracted to all yeasts, including vertebrate pathogens and other species that do not share habitats with the fly, we conclude that signaling and insect attraction is an ancient trait in yeasts, conserved over millions of years of arthropod and insect coexistence with yeasts, and is vestigial in yeasts that are not primarily associated with insects. Furthermore, coexistence of yeast and insects prior to evolution of angiosperms, overlap of signals attracting insects to yeasts and flowers, as well as functional similarities between insect–yeast interactions and insect pollination suggest to consider yeasts in the evolution of insect‐mediated pollination of flowering plants.

## CONFLICT OF INTEREST

None declared.

## AUTHOR CONTRIBUTIONS

PGB, GF, JP, and PW developed the concept and wrote the manuscript; PGB and AH ran the bioreactors; AC and ER identified the yeasts; PGB and VV performed the assays; PGB, AH, and MB performed chemical analyses; PGB and SL did the database review; AH did the BLAST search.

## DATA ACCESSIBILITY

Data used in this manuscript are present in the manuscript and its supporting information.
