# Supplementary material for: Chemical signaling and insect attraction is a conserved trait in yeasts
Source: Ecol Evol. 2018 Feb 14;8(5):2962–74. doi: 10.1002/ece3.3905 (PMC5838033; doi:10.1002/ece3.3905)
Supplement: Supplementary file 1 [file ECE3-8-2962-s001.pdf]

Appendix S1. Proteins in the reference *Saccharomyces cerevisiae* (S288C) and their corresponding orthologues in investigated yeasts

Yarrowia lipolytica (CLIB122)				*Candida albicans (WO-1)*				*Dekkera bruxelensis (AWRI 1499)*				*Kluyveromyces lactis (Y707)*				*Candida glabrata (Y475)*				*Saccharomyces cerevisiae (Y706)*				*Saccharmoyces cerevisiae (s288c)*
**Genebank ID**	**E value**	**Identities**	**Positives**	**Genebank ID**	**E value**	**Identities**	**Positives**	**Genebank ID**	**E value**	**Identities**	**Positives**	**Genebank ID**	**E value**	**Identities**	**Positives**	**Genebank ID**	**E value**	**Identities**	**Positives**	**Genebank ID**	**E value**	**Identities**	**Positives**	**Protein**
-	-	-	-	-	-	-	-	-	-	-	-	-	-	-	-	-	-	-	-	gi|392296702	6.5e-297	525/525 (100%)	525/525 (100%)	ATF1 (YOR377W)
-	-	-	-	-	-	-	-	-	-	-	-	gi|49644898	2.4e-84	171/522 (32%)	298/522 (57%)	gi|49524971	3.2e-137	247/540 (45%)	366/540 (67%)	gi|392299430	3.1e-297	535/535 (100%)	535/535 (100%)	ATF2 (YGR177C)
gi|49650080	3.4e-133	250/496 (50%)	334/496 (67%)	gi|238881848	2.3e-143	262/497 (52%)	352/497 (70%)	gi|385304822	5.1e-123	224/442 (50%)	299/442 (67%)	gi|49644672	4.6e-177	318/504 (63%)	388/504 (76%)	gi|49525724	9E-197	355/508 (69%)	419/508 (82%)	gi|392299549	4.3e-277	499/500 (99%)	500/500 (100%)	ARO8 (YGL202W)
gi|49647333	2.9e-51	132/448 (29%)	221/448 (49%)	gi|238881328	7.1e-89	171/439 (38%)	260/439 (59%)	gi|385302174	1.2e-52	132/362 (36%)	195/362 (53%)	gi|49642691	9.6e-161	292/506 (57%)	381/506 (75%)	gi|49525932	9.6e-161	292/506 (57%)	381/506 (75%)	gi|392298944	4.6e-280	513/513 (100%)	513/513 (100%)	ARO9 (YHR137W)
gi|49648423	6.8e-128	243/495 (49%)	331/495 (66%)	-	-	-	-	-	-	-	-	gi|49642640	2.4e-189	340/501 (67%)	412/501 (82%)	gi|49525613	1.8e-182	329/503 (65%)	400/503 (79%)	-	-	-	-	ALD2 (YMR170C)
-				-	-	-	-	-	-	-	-	-	-	-	-	-	-	-	-	-	-	-	-	ALD3 (YMR169C)
gi|49647250	1.5e-162	294/488 (60%)	379/488 (77%)	-	-	-	-	gi|385302426	4.6e-177	325/524 (62%)	401/524 (76%)	gi|49644235	2E-222	395/512 (77%)	457/512 (89%)	gi|49525001	1E-234	423/514 (82%)	470/514 (91%)	gi|392296700	1.6e-279	519/519 (100%)	519/519 (100%)	ALD4 (YOR374W)
-	-	-	-	gi|238882092	7.8e-175	318/499 (63%)	393/499 (78%)	-	-	-	-	gi|49641679	4E-226	398/502 (79%)	449/502 (89%)	gi|49527155	5.6e-220	400/513 (77%)	449/513 (87%)	gi|392299771	2.8e-280	520/520 (100%)	520/520 (100%)	ALD5 (YER073W)
-				-	-	-	-	-	-	-	-	gi|49644127	3E-205	364/491 (74%)	435/491 (88%)	gi|49526328	9.9e-207	361/495 (72%)	434/495 (87%)	gi|392295949	6.9e-270	500/500 (100%)	500/500 (100%)	ALD6 (YPL061W)
-	-	-	-	-	-	-	-	gi|385303323	5.3e-105	199/378 (52%)	264/378 (69%)	gi|49644238	1.3e-135	240/381 (62%)	298/381 (78%)	gi|49524727	2.2e-129	231/383 (60%)	291/383 (75%)	gi|392301243	2.7e-211	382/382 (100%)	382/382 (100%)	BDH1 (YAL060W)
-	-	-	-	gi|238879061	2.9e-97	181/394 (45%)	255/394 (64%)	-	-	-	-	gi|49644239	2E-128	230/377 (61%)	289/377 (76%)	-	-	-	-	gi|392301232	3.8e-230	412/417 (98%)	412/417 (98%)	BDH2 (YAL061W)
gi|49648146	8.6e-137	247/390 (63%)	300/390 (76%)	-	-	-	-	gi|385302974	2.3e-143	257/372 (69%)	305/372 (81%)	gi|49640582	1.1e-173	315/383 (82%)	337/383 (87%)	gi|49525675	4.9e-173	308/372 (82%)	334/372 (89%)	gi|392299022	4.1e-217	393/393 (100%)	393/393 (100%)	BAT1 (YHR208W)
-	-	-	-	gi|238882204	5.6e-133	248/371 (66%)	287/371 (77%)	-	-	-	-	-	-	-	-	-	-	-	-	gi|392298577	6.7e-208	376/376 (100%)	376/376 (100%)	BAT2 (YJR148W)

Orthologs are identified by their unique Genebank ID. The E value indicates the statistical significance of each alignment. The lower the E value, the more significant is the hit, and the less likely it is to have occured by chance. Identities is the ratio (and percentage) of identical aminoacids and the subject length for each alignment. Positives is the ratio (and percentage) of conservative substitutions and the subject length for each alignment, as defined by the substitution matrix (BLOSUM62).
